# Motor Competence in Autistic Children with Attention-Deficit Hyperactivity Disorder

**DOI:** 10.3390/children11121518

**Published:** 2024-12-14

**Authors:** Jadiane Dionisio, Cristina dos Santos Cardoso de Sá, Susana Lúcio, Gabriela Neves de Almeida, Rita Cordovil

**Affiliations:** 1 Faculdade de Educação Física e Fisioterapia, Universidade Federal de Uberlândia, Uberlândia 38400-678, Brazil; 2 Departamento de Fisioterapia, Escola Superior se Saúde de Alcoitão, 2649-506 Alcabideche, Portugal; cristina.sa@uol.com.br; 3 CADIn-Neurodesenvolvimento e Inclusão, 2750-782 Cascais, Portugal; 4 Escola de Saúde e Desenvolvimento Humano, Comprehensive Health Research Centre, Universidade de Évora, 7002-554 Évora, Portugal; 5 CIPER, Faculdade de Motricidade Humana, Universidade de Lisboa, 1499-002 Cruz Quebrada-Dafundo, Portugal; ritacordovil@fmh.ulisboa.pt

**Keywords:** autism spectrum disorder, motor skills, neurodevelopmental disorders, exercise test

## Abstract

Background/Objectives: Children with autism exhibit deficits in fundamental motor skills, which are intensified when associated with attention-deficit/hyperactivity disorder (ADHD). Objective: To correlate motor competence in children with autism, autism levels according to the CARS scale, and the association with ADHD. Methods: This cross-sectional study assessed motor competence using the Motor Competence Assessment (MCA), the severity of Autism Spectrum Disorder by Childhood Autism Rating Scale (CARS), and the presence of clinical signs for ADHD using Swanson, Nolan, and Pelham (SNAP-IV) questionnaire. A total of 68 children were recruited; however, 23 were not included due to non-acceptance and/or were excluded because of associated pathologies, high autism severity. Consequently, 45 children (11 with ADHD) between the ages of 5 and 11 (Mean: 8.15; ±1.75) with 83.72% being male. Results: Multiple linear regression revealed a significant inverse correlation between MCA scores and raw CARS data (*p* = 0.01), indicating that greater autism severity is associated with lower motor competence. The correlation was also observed in CARS classifications, with higher autism severity corresponding to lower motor percentiles (*p* = 0.05). However, the correlation between the presence of ADHD and motor competence in children with ASD did not reach significance. Overall, a significant correlation was observed (*p* = 0.006) when analyzing all variables (CARS scores, SNAP scores, and CARS classification). Conclusion: Therefore, it is believed that children with autism exhibit deficits in functional motor skills, with severity impacting motor competence. The findings underscore the need for systematic motor skill assessments in children with autism, emphasizing the importance of monitoring and intervention in this aspect of their development.

## 1. Introduction

Motor skills, along with executive function, are essential for learning and adaptation in social and school environments, promoting a child’s development [1]. Fundamental motor skills are primarily manifested through actions involving balance, locomotion, and object manipulation [2,3], improving with age, with object manipulation tasks being more pronounced in males [4].

Deficits in fundamental motor skills (balance, gross motor, fine motor, coordination, and others) tend to be present in autism spectrum disorder (ASD), especially during childhood, due to alterations in pyramidal, extrapyramidal, supplementary, and cerebellar neural pathways [5,6]. In addition to neural issues, the association of ASD with other disorders, whether intellectual [7], social or attention-deficit/hyperactivity disorder (ADHD) [8], can also contribute to alterations in fundamental motor skills.

In relation to intelligence, the association between gross motor and fine motor changes with the intelligence quotient (IQ) occurs both for children with autism with high and low IQ, and when comparing the groups, the one with low IQ will show greater changes gross motor skills compared to children with high IQ. However, praxis correlates with the degree of autism, regardless of intellectual factors [9].

In the same sense, there is an association between attention deficit and impaired motor skills in children with high-functioning ASD, since the central controls of orientation, attention, and alertness influence motor learning and reaction time, these being altered in this population [10].

Attention-deficit/hyperactivity disorder (ADHD) strongly influences behavioral, cognitive [11], and motor actions in children with ASD, leading to compromised motor competence, such as manual actions. This is attributed to difficulties in inhibition and control of motor response [12].

Motor disorders present in children with ASD, associated with deficiencies in emotional, behavioral, and social skills, can result in negative impacts, leading to reduced participation in physical activities and a possible increase in comorbidities. There is a need to expand the scope of studies concerning motor impairments, an aspect relatively scarce in the scientific literature [13,14].

Children with autism present deficits in coordination and balance; however, there has been little study on the correlation between the level of autism and motor competence, as well as the association of autism with other mental disorders, such as ADHD. It is believed that a child with ASD associated with ADHD will exhibit lower motor competence, which characterizes the performance of motor skills through movement quality, coordination, and motor control [15], compared to ASD without ADHD. Therefore, the objective of this study is to correlate the motor competence of children diagnosed with autism associated with ADHD, as well as to verify if the level of autism is related to low motor performance.

## 2. Materials and Methods

This cross-sectional study evaluating the motor competence of children with autism, was approved by the Ethics Committee (registration number: 01-2023, approval: March 2023) and informed consent was obtained from all participants, following the recommendations of the Helsinki Declaration. Furthermore, all participants/guardians received, at the end of the study, the participation report containing all the information and actions of the study, along with the results and conclusions.

The sample size calculation was based on the most recent study identifying the prevalence of ASD in the central region of the country where this study was conducted. Rasga et al. [16] evaluated 13,690 children in 173 schools in the region, identifying 55 children with ASD between the ages of 7 and 9. Since the objective of this study was to include school-age children in childhood, the calculation was performed for a homogeneous sample, with a 5% margin of error and a 95% confidence level, resulting in a suggested sample of 46 children with an ASD diagnosis.

### 2.1. Participants

The inclusion criteria covered children of both sexes, various ethnicities, and social classes, aged 5 to 11 years old with a medical diagnosis of ASD levels I and II according to DSM-5TR [17], residing in Portugal. Exclusion criteria were associated neurological pathologies, non-signing of the consent form, and absence and/or non-execution of more than 60% of the motor competence assessment instrument.

A total of 68 children were recruited. Among them, 11 children were not included due to non-acceptance of participation or prolonged absence from the institution, and 12 children were excluded due to associated pathologies, high autism severity, and/or absence on the day of evaluation. Consequently, 45 children between the ages of 5 and 11 (Mean: 8.15; ±1.75) were evaluated, with an average weight of 32.54 (±13.01) and an average height of 132.66 cm (±14.22). Of these, 83.72% were male, representing various ethnicities, races, and social classes. All children had an adequate level of understanding of the MCA activities, underwent therapy sessions one to two times a week, and were enrolled in regular school, residing in different cities. Of the 45 children with ASD classified as level I and II according to the DSM-5TR, 11 (24.44%) had ADHD associated, including 3 with ADHD (6.67%), 3 with ADHD inattentive type (6.67%), and 5 with ADHD hyperactive type (11.11%). Based on the CARS scores, 43 (95,55%) children were categorized as mild to moderate autism and 2 (4,44%) as severe autism.

### 2.2. Instruments

For the assessments, three different scales were employed, addressing the evaluation of motor competence, classification of the severity of ASD, and the presence of clinical signs for ADHD.

#### 2.2.1. MCA—Motor Competence Assessment [18,19]

It is a quantitative instrument that assesses motor competence through six tasks involving three components: stability: (i) shifting platforms—individuals move laterally for 20s using two wooden platforms; and (ii) Jumping Sideways —subjects jump sideways with both feet together, clearing a small wooden beam for 15s; locomotor (i) Shuttle Run—running at maximum speed between the starting line and a line placed 10 m away, picking up a wooden block, running backward, and placing the block beyond the starting line twice; and ii) standing long jump—jumping simultaneously with both feet as far as possible; and manipulative: (i) ball kicking velocity—kicking a soccer ball against the wall; and (ii) ball throwing velocity—throwing a tennis ball at a target with maximum force. In both tests, peak speed is measured in m/s using a Bushnell Radar speed gun (model 10-1911).

The MCA is a new and unique instrument for assessing motor competence across the lifespan. Over the past few years, the research team that created it has completed various stages of instrument validation. In the first phase, the structural validity of the MCA construct was established, defined by six tests representing three major components of motor competence: locomotor; stability; and manipulative or object control. Subsequently, the normative distribution of the MCA tests from childhood to early adulthood, up to 23 years old, was tested and presented for the Portuguese population [18,19].

#### 2.2.2. CARS—Childhood Autism Rating Scale [20]

The CARS is a brief scale that aids in the identification of children with ASD through 15 items, including the following: personal relationships, imitation activities, emotion, bodily response, visual and auditory response, as well as changes in taste, smell, object use, adaptation to changes, verbal and non-verbal communication, and signs of fear, nervousness, activity level, and intellectual consistency. At the end of the scale, based on the assigned score (ranged from a low of 15 to a high of 60), the child’s clinical condition is identified as follows: no autism (score < 30), mild to moderate autism, and severe autism (score >38). The CARS scale is a sensitive scale for identifying ASD, but it does not provide specific details, so it is recommended to use it in conjunction with another scale [18]. Therefore, we used the CARS scale alongside the American Psychiatric Association (DSM-5TR) to classify participants into the three support levels proposed by the DSM-5TR [17].

#### 2.2.3. SNAP-IV—Swanson, Nolan and Pelham [21]

The SNAP-IV is a questionnaire constructed based on the Diagnostic and Statistical Manual of Mental Disorders (DSM-IV) with the aim of evaluating the symptoms of ADHD. The instrument consists of 18 items, scored as “Not at all”, “Just a little”, “Quite a bit”, or “Very much”. The first nine items suggest symptoms of attention deficit, with a recommendation of attention deficit for children scoring six or more items as “Quite a bit” or “Very much”. The remaining nine items relate to hyperactivity, with a suggestion of hyperactivity for children scoring six or more items as “Quite a bit” or “Very much”. The instrument can be used by healthcare professionals, teachers, and even caregivers. It is important to note that the instrument does not diagnose ADHD, but rather, it identifies symptoms. The SNAP-IV scale has high sensitivity for screening, making it suitable for use in clinical trials [22].

### 2.3. Procedure

Data collection began by contacting the institutions and parents/guardians to explain the procedure and obtain the signed consent form. After this process, individual assessment dates for each child were scheduled.

The assessments took place at the institution itself during the therapy hours or immediately after therapy sessions. The child’s therapist always monitored the collection. The duration of each individual assessment ranged from 20 to 30 min.

Initially, the researcher introduced herself to the child, collected data on date of birth, weight, and height and explained the MCA test to assess motor competence. After the explanation, the child tried out the test and then performed the practice, which was recorded by the researcher on a specific form. Two physiotherapists previously trained and with experience in the area of neurodevelopment collected the data.

Once the motor competence assessment was completed, the degree of autism was assessed using the CARS scale and ADHD assessment was carried out using the SNAP-IV questionnaire. If the child felt tired, irritated, or disinterested, the test was interrupted and restarted after a few minutes depending on the child’s interest. If this was not possible, a new collection date was scheduled. Data collection was conducted by two researchers who had undergone prior training, including a pilot test, and had achieved a satisfactory level of reliability.

The data were recorded on designated forms and then transferred to the computer using the SPSS 28.0 statistical package. A multiple linear regression analysis and the statistical test of correlation coefficient, with a significance level of 0.005, were performed to identify correlations between continuous variables and categories, namely MCA score, raw CARS score, ASD classification by CARS, and the presence of ADHD by SNAP-IV, respectively.

## 3. Results

**Figure 1:**
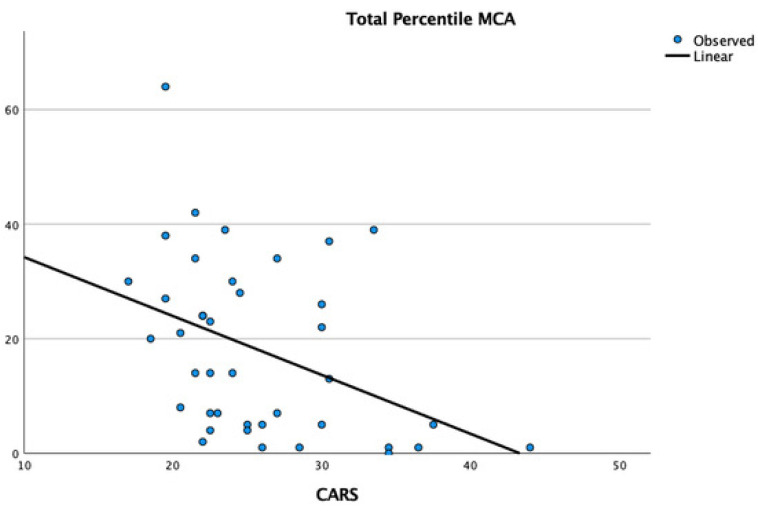
Graphical representation of the correlation between MCA percentile and raw scores from the CARS Scale.

Multiple linear regression identified a significant inverse positive correlation between the MCA score and the raw CARS data (t: −2.67, *p* = 0.01), indicating that a higher CARS score is associated with lower motor competence (Figure 1).

**Figure 2:**
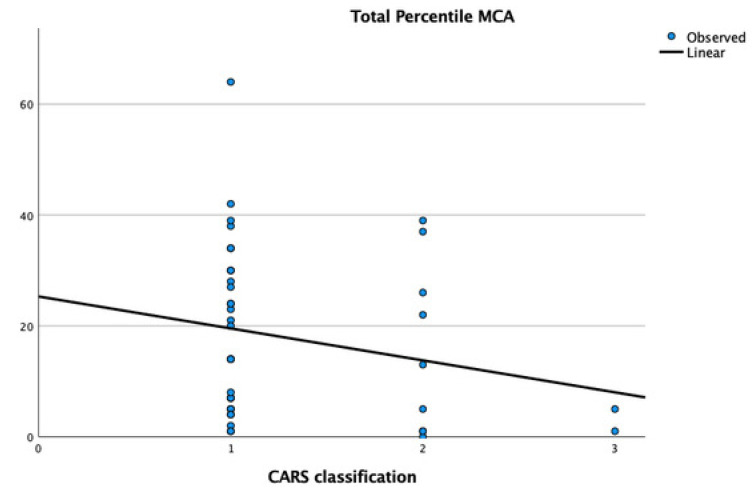
Graphical representation of the correlation between MCA percentile and ASD severity classification, with 1-mild, 2-moderate, 3-severe.

Regarding the classification of CARS, an inverse correlation with motor competence was also observed (t: 1.99, *p* = 0.05), indicating that children with higher degrees of ASD have lower motor percentiles in the MCA (Figure 2).

**Figure 3:**
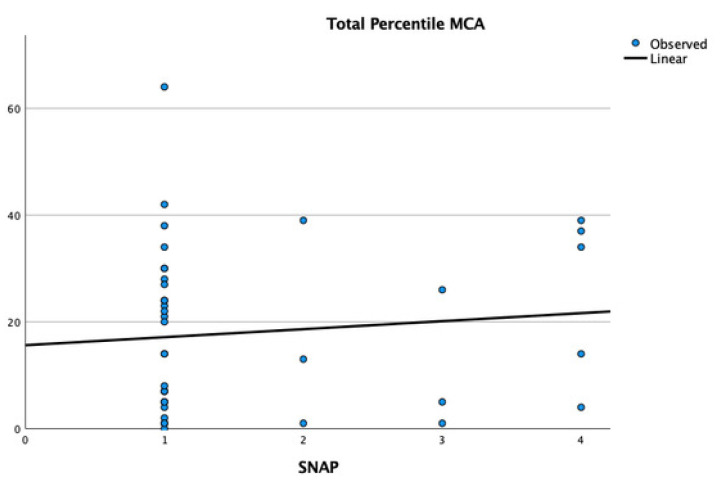
Graphical representation of the correlation between MCA percentile and the presence of ADHD in children with ASD, with 1-children without ADHD; 2-children with ADHD, 3-children with inattention, and 4-children with hyperactivity.

Finally, the correlation between the presence of ADHD and motor performance in children with ASD did not show significance (t: 1.58, *p* = 0.12) (Figure 3).

In the general analysis, when correlating all variables (total CARS score, SNAP score, and CARS classification), a significant correlation was observed (F: 885.870, Z: 4.923, *p*: 0.006).

## 4. Discussion

The objective of this study was to correlate the motor competence of children diagnosed with autism associated with ADHD, as well as to verify if the level of autism is related to low motor performance. The hypothesis that a higher level of ASD is related to a decrease in motor competence was confirmed, while the correlation between ASD and ADHD with motor competence was refuted.

Autism spectrum disorder can be classified based on the level of support needed, corresponding to the severity of symptoms. Level 1 is considered mild, indicating more independent children. Level 2 is moderate, signifying children with a substantial need for support. Level 3 is severe, indicating a significant need for support [17]. The symptoms are related to social communication, interaction, and behavior [23].

Behaviors change is observed in children with ASD across all three levels, with stereotyped, repetitive behavior, self-injury, and social interaction deficits being more intense in Level 3 ASD [24]. Just as behavioral, adaptive, cognitive, and social alterations vary across different levels of ASD, motor skills may also exhibit variability. This aligns with the findings of this study, where the severity level of ASD, as assessed by CARS (classification and total score), is directly associated with lower motor competence.

The correlation between motor disorders and social communication is explained by mirror neuron system dysfunction [25]. Additionally, cerebellar and sensorimotor alterations contribute, with children exhibiting higher levels of ASD demonstrating lower motor abilities, especially in fine motor skills, visuomotor tasks, and perception–action tasks [26]. Another justification found in the literature relates to cortical hemispheric changes. Children with severe ASD exhibit lower cerebral complexity with a predominance of the right hemisphere, particularly in the right frontal, left central, and bilateral parietal regions [27].

In recent years, advancements in understanding motor impairments in children with ASD, regardless of gender [28], have led the scientific community to advocate for the inclusion of motor skill assessment as a clinical specifier within the Diagnostic and Statistical Manual of Mental Disorders (DSM). This recommendation aims to encourage professionals to regularly assess and address the motor components of children with ASD, emphasizing the importance of evaluating motor competence [29,30,31].

When comparing the fundamental motor skills in autism to those with ADHD and typical children, using the Test of Gross Motor Development, Pan et al. [32] observed that all the children with ASD and ADHD exhibited lower motor performance compared to non-ASD and non-ADHD children. The authors suggest that distinct mechanisms, such as increased white matter in the pre-motor cortex, deficits in mirror neurons, and sensory processing in children with ASD, as well as reduced brain volume in the right frontal region, corpus callosum, and cerebellum in those with ADHD, contribute to impairments in motor performance [33]. This reinforces the need for professionals to expand their focus on the motor performance of children with ASD and ADHD.

With the aim of assessing motor skills and imitation in children with ASD, ADHD, and typical development, Biscaldi et al. [34] observed that both the ASD and ADHD groups exhibited lower motor abilities compared to typical children. However, regarding imitation, deficits were found only in children with ASD. The authors highlighted motor alterations related to the quality and speed of movement, emphasizing the differences between groups in terms of balance. Dynamic balance deficits were more pronounced in children with ASD, while static balance deficits were more evident in those with ADHD.

Although children with ADHD present balance deficits [35], and contrary to expectations, no correlation was observed between motor competence deficits in children with ASD associated with ADHD. It is believed that this result may be due to the low number of children with ADHD (3 children), attention deficit (3 children), and hyperactivity (5 children). Ramos-Sánchez et al. [36], in their investigation of motor variability in children with ASD associated with intellectual impairments and ADHD, observed that children with ASD associated with ADHD had lower total scores on the Motor Assessment Battery for Children-2 (MABC-2) compared to children with ASD without ADHD. The authors suggested that the combination of these disorders leads to alterations in memory, attention, and executive functioning, resulting in lower motor performance. Despite these findings, few studies correlate the motor competence of ASD associated with ADHD, highlighting the need to expand scientific research on this topic.

## 5. Limitations

Although the sample size calculation of the study matches the number of children evaluated, since this study was conducted based on the latest epidemiological analysis of ASD in the central region of the country, unfortunately, the group of children with ASD associated with ADHD was small, which is a limiting factor. It is suggested that further studies with a larger group of children with ASD associated with ADHD be conducted to verify and confirm whether these results will be similar.

## 6. Conclusions

In conclusion, children with ASD exhibit deficits in functional motor skills, with these being more prominent in children with higher levels of ASD. Although no statistical difference was observed regarding the motor competence of children with ASD associated with ADHD, it is important to emphasize the need for further studies, especially comparing ADHD subtypes (inattentive, hyperactive–impulsive, and combined) and motor competence.

Training of fundamental motor skills is essential to improving an individual’s motor performance, as both single- and multiple-task actions are crucial for daily life.

One way to support this process and monitor the progress of children with ASD is through the use of standardized scales that focus on fundamental motor skills for tracking the motor competence of children with ASD, with or without ADHD. This can help guide treatment and provide appropriate stimuli for the exercises performed.

## Data Availability

The data presented in this study are available on request from the corresponding author. The data are not publicly available due to privacy or ethical restrictions.
